# mNFE: microbiome network flow entropy for detecting pre-disease states of type 1 diabetes

**DOI:** 10.1080/19490976.2024.2327349

**Published:** 2024-03-21

**Authors:** Rong Gao, Peiluan Li, Yueqiong Ni, Xueqing Peng, Jing Ren, Luonan Chen

**Affiliations:** a School of Mathematics and Statistics, Henan University of Science and Technology, Luoyang, China; b Big Data Institute, Central South university, Changsha, China; c Longmen Laboratory, Luoyang, Henan, China; d Department of Microbiome Dynamics, Leibniz Institute for Natural Product Research and Infection Biology, Hans Knöll Institute, Jena, Germany; e Cluster of Excellence Balance of the Microverse, Friedrich Schiller University Jena, Jena, Germany; f Key Laboratory of Systems Biology, Institute of Biochemistry and Cell Biology, Center for Excellence in Molecular Cell Science, Chinese Academy of Sciences, Shanghai, China; g Key Laboratory of Systems Health Science of Zhejiang Province, Hangzhou Institute for Advanced Study, University of Chinese Academy of Sciences, Hangzhou, China; h West China Biomedical Big Data Center, West China Hospital, Sichuan University, Chengdu, China

**Keywords:** Tipping point, dynamic network biomarker (DNB), gut microbiome, Type 1 diabetes (T1D), network flow entropy (NFE)

## Abstract

In the development of Type 1 diabetes (T1D), there are critical states just before drastic changes, and identifying these pre-disease states may predict T1D or provide crucial early-warning signals. Unlike gene expression data, gut microbiome data can be collected noninvasively from stool samples. Gut microbiome sequencing data contain different levels of phylogenetic information that can be utilized to detect the tipping point or critical state in a reliable manner, thereby providing accurate and effective early-warning signals. However, it is still difficult to detect the critical state of T1D based on gut microbiome data due to generally non-significant differences between healthy and critical states. To address this problem, we proposed a new method – microbiome network flow entropy (mNFE) based on a single sample from each individual – for detecting the critical state before seroconversion and abrupt transitions of T1D at various taxonomic levels. The numerical simulation validated the robustness of mNFE under different noise levels. Furthermore, based on real datasets, mNFE successfully identified the critical states and their dynamic network biomarkers (DNBs) at different taxonomic levels. In addition, we found some high-frequency species, which are closely related to the unique clinical characteristics of autoantibodies at the four levels, and identified some non-differential ‘dark species’ play important roles during the T1D progression. mNFE can robustly and effectively detect the pre-disease states at various taxonomic levels and identify the corresponding DNBs with only a single sample for each individual. Therefore, our mNFE method provides a new approach not only for T1D pre-disease diagnosis or preventative treatment but also for preventative medicine of other diseases by gut microbiome.

## Introduction

The majority of Type 1 diabetes (T1D) patients have autoimmune disease that targets pancreatic islet beta cells and arises from complex interactions among genetic elements, patient exposures, and the gut microbiome, usually occurring in children and young adults. And only a minority of patients with T1D have non-autoimmune pathogenesis.^1–3^ Destruction of 90% of beta cells is a critical point at which clinical manifestations emerge.^4^ Because of the early onset and chronicity of this disease, the detection of critical states in the development of T1D is of great importance. The development of biological processes is often viewed as a time-dependent nonlinear dynamical system or network involving non-smooth or abrupt state transitions.^5,6^ Similarly, T1D development with a critical transition point can be generally divided into three states, i.e., normal/healthy state, pre-disease/critical state, and disease state. A normal state is a relatively stable or healthy state before the transition. The pre-disease state or tipping point is the limit of the normal state just before the imminent drastic transition, which is reversible to the normal state if appropriately treated. If not appropriately treated, the system will develop into disease state, which is another stable state and irreversible to the normal state.^5,7–9^ Thus, timely detection of the pre-disease or critical state is of great importance for early diagnosis and treatment. However, it is challenging to detect the tipping point or critical state because a normal state and its critical state are similar in terms of both phenotype and gene expression, which leads to the failure of traditional biomarkers in detecting the critical state.

To address this important challenge, the concept of the dynamic network biomarker (DNB), defined by three statistical conditions, has been developed to quantify the critical state (or called “WeiBing” in traditional Chinese medicine) or provide early-warning signals of the disease state during disease development.^8^ DNB members consist of a set of molecules, genes, or proteins that are strongly fluctuating and also strongly correlated, i.e. they can be characterized as “critical collective fluctuations” in terms of variables in contrast to “critical slowing down” in terms of states.^8^ Unlike traditional biomarkers, the concentrations of DNB molecules fluctuate collectively when approaching the critical state, rather than exhibiting constant values or random fluctuations.^10^ In particular, when the system is near a bifurcation point or tipping point, a dominant group or DNB appears, and the members of the dominant group or DNB satisfy the following three conditions: the correlation between DNB members rapidly increases, the standard deviation for each DNB member drastically increases, and the correlation between one DNB member and any non-DNB molecule decreases.^7,8^

Increasing evidence suggests that the gut microbiota is involved in the pathogenesis of T1D.^11^ The gut microbiota plays an important role in the regulation of autoimmunity and tolerance.^12–15^ It has been proposed that altered intestinal microbiota may impact T1D pathogenesis by increasing gut permeability, facilitating intestinal inflammation, and disturbing immunological maturation.^16–19^ Most previous studies have identified key bacteria as biomarkers based on variation in abundance between healthy and diseased groups.^20,21^ However, the existing studies have mainly concentrated on revealing the role of gut bacteria in the development of T1D or identifying its disease state, which may fail in identifying its pre-disease state on a single-sample basis.

A number of DNB-based computational methods have been developed to detect the critical states of diabetes using gene expression data. The early-warning signals of T1D and its leading biomolecular networks have been detected and used to mark the time period just before the drastic deterioration to diabetes.^22,23^ The degree matrix network entropy method can detect the critical states of type 2 diabetes mellitus based on a sample-specific network.^24^ However, these methods are mainly based on gene expression data and suffer from unsatisfactory effectiveness and robustness due to highly noisy omics data, especially on the basis of a single sample. Unlike gene expression data, gut microbiome sequencing data can be noninvasively collected from stool samples, so they are more easily accessible. The collected metagenomic data contain phylogenetic information at various taxonomic levels, which can help us detect early-warning signals of T1D, thus improving the robustness and effectiveness of the mNFE method. Therefore, developing an effective and robust method to detect the critical state of the gut microbiome in T1D with only a single sample from an individual is an important and challenging task.

To solve this problem, we proposed a novel method, i.e., microbiome network flow entropy (mNFE), to detect the critical state of T1D based on solely a single sample of gut microbiota from an individual. First, we constructed a microbial association network by using the neighborhood selection (mb) method based on SParse InversE Covariance Estimation for Ecological Association Inference (SPIEC-EASI). Then we calculated the NFE by extracting the local- or sub-network from the sample-specific association network. Finally, the global/whole network disturbance caused by a single case sample was quantified by mNFE. By transforming the microbial profile from sequencing data into NFE, the mNFE method provides a new way to detect the critical state based on a single sample from any individual. Our proposed method has the following advantages: (i) The stool samples needed for human gut microbiome sequencing are collected noninvasively and can be sampled repeatedly. Gut microbiome sequencing data contain different levels of phylogenetic information that can be utilized to detect the tipping point in a reliable manner, thereby providing more accurate and effective early-warning signals. (ii) The mNFE method reliably quantifies network fluctuation, that is, the collective molecule fluctuation induced by each single sample for a set of given reference samples, thereby reducing the noise and enhancing the robustness by mining dynamical and high-dimensional information of T1D omics data. (iii) With the mNFE method, we can successfully detect the critical states at various taxonomic levels and identify the corresponding DNBs with only a single sample for each individual, both of which contribute to the effectiveness and robustness of this method. (iv) The mNFE method is a model-free and data-driven method, which can achieve the detection of early-warning signals for T1D based on a single sample from an individual. (v) Based on the mNFE method, some high-frequency species that are closely related to the unique clinical characteristics of autoantibodies were observed at the four levels, including Glutamic Acid Decarboxylase (GADA), insulinoma antigen-2 (IA-2A), insulin (IAA) and Zinc Transporter 8 (ZnT8A) levels. And some non-differential ‘dark species’ that play important roles in T1D development were identified. Network connectivity and fragility were measured to illustrate the potential interactions among species in control, seroconversion, and T1D case groups.

The mNFE method is capable of detecting the tipping point before the onset of seroconversion and T1D symptoms at different taxonomic levels and identifying the corresponding DNBs, both of which enhance the effectiveness and accuracy of this method. The critical states of all symptomatic subjects were detected before symptoms appeared based on the high-frequency species. The numerical simulation demonstrated the robustness of mNFE. Some identified high-frequency species were found to be associated with the clinical features of autoantibodies at the four levels. We also revealed the ‘dark species’ that are related to the development of T1D.We showed that the connectivity and robustness of the microbial association network in the control and seroconversion groups were significantly stronger than in the T1D case group.

## Results

To illustrate how mNFE works, we first applied it to a simulated dataset, and then to T1D. The proposed method successfully detected the early-warning signals of critical transition into an irreversible after-transition state, which validated the effectiveness of our method in identifying the tipping point just before the critical transition.

### Validation based on numerical simulation

To validate the proposed mNFE method, we employed a regulatory network with eight nodes, which was inspired by previous works.^25,26^ A model of gene regulatory network of Michaelis – Menten or Hill form^27,28^ is typically used to study genetic regulations including transcription and diffusion processes,^29,30^ nonlinear biological processes, and other gene regulatory activities.^31,32^ To transform the simulation model into a complete time series, the numerical data were generated from the model network with a parameter p varying from − 0.5 to 0.25.

**Figure 1.**
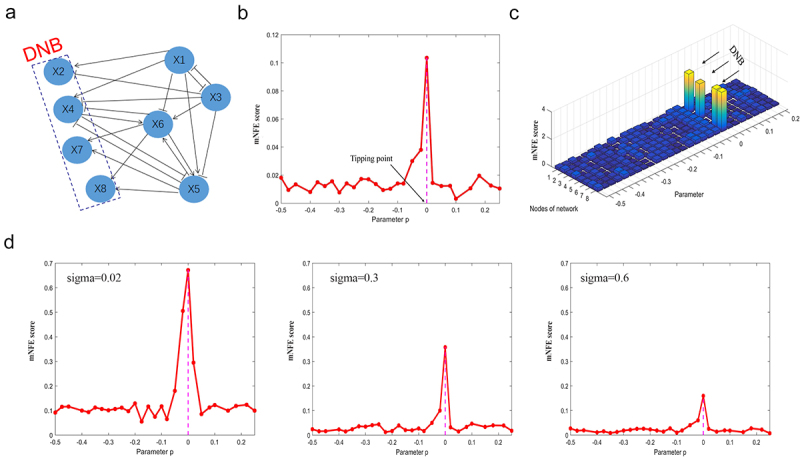
The validation of the mNFE method on a simulation dataset.

The gene regulatory network of eight nodes with positive or negative relationships is demonstrated in Figure 1a. Constructing the gene regulatory network based on the generated data with a set of parameters, we performed the numerical experiment. As shown in Figure 1b, the sudden increase of global mNFE score represents the imminent early-warning signals when the system is near the bifurcation parameter value *p* = 0. Thus, the significant increase of mNFE score implies the upcoming critical transition at *p* = 0. To better illustrate the distinct dynamics between the normal state and the critical state, the mNFE landscape of the eight nodes is illustrated in Figure 1c. In particular, when the system is far away from the tipping point, the mNFE score of each node is at a low level; when the system approaches the tipping point *p* = 0, the mNFE score increases sharply. In addition, we have analyzed the stability and robustness of the mNFE method under different noise strengths in Figure 1d. With the increase of noise strength, the mNFE method remained stable in providing the early-warning signals of critical transition, which shows that the mNFE method is stable and robust. The simulation and calculation details are presented in the Supplementary Material, Figure S1.

(A) The model of an eight-node network, in which the arrow represents positive regulation, and the blunt line represents negative regulation. (B) The curve of the mNFE score based on the gene regulatory network. (C) The landscape of the mNFE scores for eight nodes. (D) The robustness of the mNFE method under different noise strengths.

### Critical states of T1D based on the mNFE

#### Critical states of T1D at the different taxonomical levels

**Figure 2.**
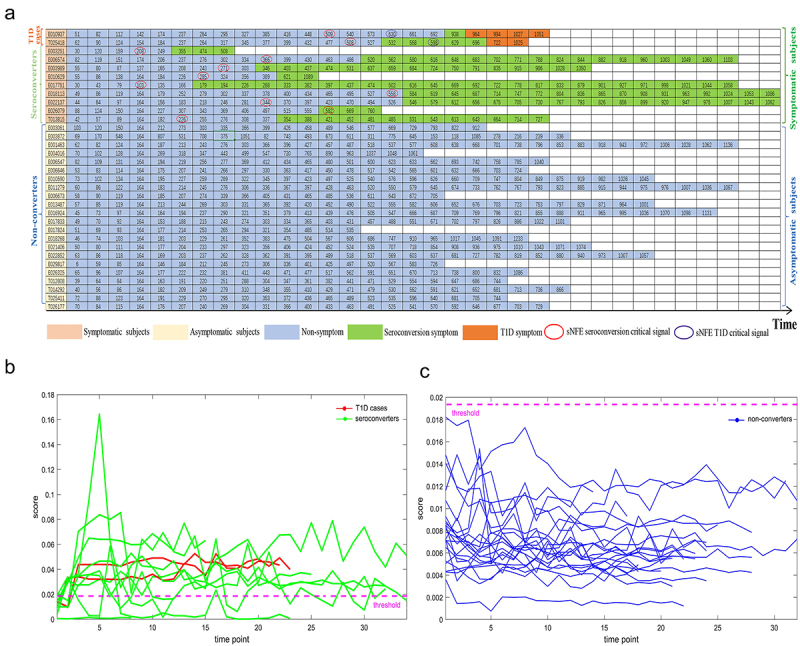
The identification of critical states for seroconversion and T1D based on mNFE at the overall taxonomic level.

**Figure 3.**
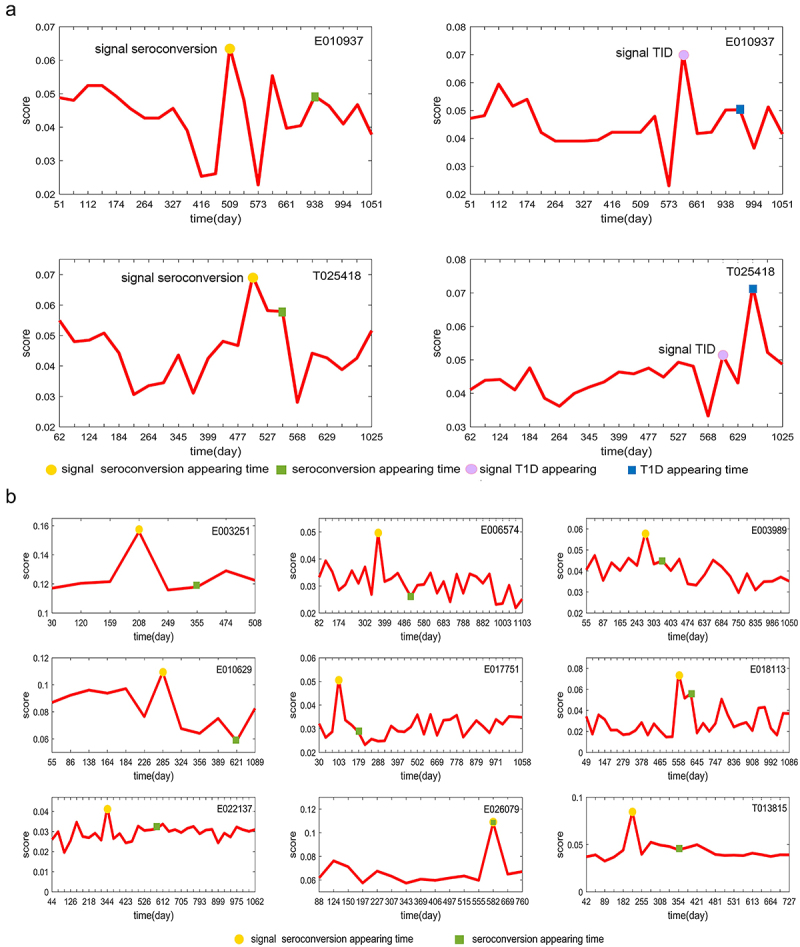
The curves of mNFE scores for all symptomatic subjects based on their respective DNBs at the overall taxonomic level.

We then applied the mNFE method to a previously published longitudinal dataset of T1D.^33^ As shown in Figure 2a, there were 33 subjects/infants, 11 of whom seroconverted to serum autoantibody positivity, and among those 11, four developed T1D. The 16S rRNA sequencing was performed on stool samples with a median of 23 unique samples per individual (minimum 8, maximum 34), and a full operational taxonomic unit table was created. We divided the 33 subjects into a symptomatic group containing T1D cases and seroconverters, and an asymptomatic group containing non-converters. Since the number of time points with sequencing data from two of the four T1D cases was insufficient, we could only detect the tipping point of seroconversion in these two individuals, thus we put their results of early-warning signals together with the seroconverters. As shown in Figure 2b,c, the global mNFE score curves of T1D and seroconverted individuals showed obvious signals, while the score curves of nonconverters showed no significant change. The top 10% features with the largest mNFE scores at the critical state were regarded as DNBs.^26^ For 11 symptomatic subjects, the specific mNFE scores for their DNBs are shown in Figure 3a,b. These scores demonstrated that early-warning signals for T1D and seroconverted individuals preceded the onset of symptoms, expect for the individual E026079 where the early-warning signal co-occurred with symptoms.

(A) Table of mNFE diagnoses and clinical diagnoses for all subjects. (B) Line chart for early-warning signals in all symptomatic subjects. The red curve represents the mNFE scores for two T1D subjects. The green curve represents the mNFE score for nine seroconverters. (C) Line chart for early-warning signals in all asymptomatic subjects. The blue curve represents the mNFE score for 22 non-converters.

(A) The individual mNFE score curves of two T1D subjects based on their respective DNBs. (B) The individual mNFE score curves of nine seroconverters based on their respective DNBs. The yellow circle represents the pre-seroconversion state identified by the mNFE score, and the green square represents the time point of seroconversion onset (clinically observed). The purple circle stands for the tipping point or the critical state identified by the mNFE score, and the blue square denotes the time point of T1D onset (clinically observed).

In addition, to improve the effectiveness of the mNFE method, we detected the early-warning signals of seroconversion and T1D at the genus and species levels, respectively. The results on the real dataset indicated that the critical states could be successfully detected before the onset of seroconversion and T1D at the genus and species levels. The details for the detection of tipping point at the species and genus levels are provided in the Supplementary Material, Figure S2 and S3.

### Overview of the detection of critical states at different taxonomic levels

Moreover, we overviewed the tipping point detected for all individuals at three different levels, i.e., the overall taxonomic level, genus level, and species level. As shown in Table 1, for 11 symptomatic subjects, the 11/11 mNFE prediction was in advance of the symptom appearing time at all three different levels, and there were no wrong signals for 22 asymptomatic individuals, reflecting the high effectiveness and accuracy of the mNFE method in detecting the critical state on an individual basis.

**Table 1.** Overview of the detection of early-warning signals based on the mNFE at different taxonomic levels.

					Overall taxonomic level	Genus level	Species level
	Individual		Seroconversion onset	T1D onset	Early-warning signal		
Symptomatic individuals (Sym)	T1D cases	E010937	938day	964day	509&630day	385&661day	630&692day
		T025418	532day	722day	508&598day	508&629day	477&568day
		E003251	355day	\	208day	208day	208day
		E006574	520day	\	366day	366day	276day
	Seroconverters	E003989	346day	\	271day	271day	208day
		E010629	621day	\	285day	285day	285day
		E017751	179day	\	103day	103day	135day
		E018113	584day	\	558day	495day	302day
		E022137	546day	\	344day	344day	370day
		E026079	582day	\	582day	227day	515day
		T013815	354day	\	226day	182day	182day
Asymptomatic individuals (Asym)	Non-converters	E003061	Asym	Asym	No	No	No
		E003872	Asym	Asym	No	No	No
		E001463	Asym	Asym	No	No	No
		E004016	Asym	Asym	No	No	No
		E006547	Asym	Asym	No	No	No
		E006646	Asym	Asym	No	No	No
		E010590	Asym	Asym	No	No	No
		E011279	Asym	Asym	No	No	No
		E006673	Asym	Asym	No	No	No
		E013487	Asym	Asym	No	No	No
		E016924	Asym	Asym	No	No	No
		E017833	Asym	Asym	No	No	No
		E017824	Asym	Asym	No	No	No
		E018268	Asym	Asym	No	No	No
		E021406	Asym	Asym	No	No	No
		E022852	Asym	Asym	No	No	No
		E029817	Asym	Asym	No	No	No
		E026325	Asym	Asym	No	No	No
		T012808	Asym	Asym	No	No	No
		T014292	Asym	Asym	No	No	No
		T025411	Asym	Asym	No	No	No
		T026177	Asym	Asym	No	No	No
	The signals detected for Symptomatic individuals (The accuracy rate of identification)				11/11	11/11	11/11
	Is there any wrong signal for Asymptomatic individuals?				No	No	No

### Diversity analysis and accuracy analysis of mNFE

**Figure 4.**
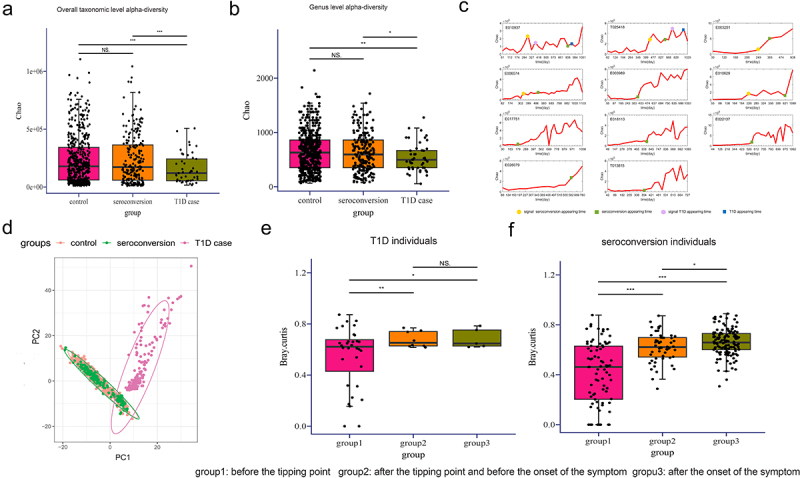
Diversity analysis and accuracy analysis of mNFE.

In order to reveal the microbial richness and internal diversity of each individual sample, we firstly calculated and compared microbiome alpha diversity in the control, seroconversion and T1D case groups. We observed significant differences in alpha diversity (Chao) between T1D and the other two groups (*p* <.05, Wilcoxon rank-sum test) (Figure 4a,b). The alpha diversity of control individuals and seroconversion individuals were higher than that of T1D cases, which is consistent with previous studies.^33^ Moreover, we compared mNFE with the Chao index for early-warning signals in the seroconversion and T1D individuals. We used the score indices, i.e., $\mathrm{mNF}E^{T}>1.5\mathrm{mNF}E^{T-1}\mathrm{andCha}o^{T}>1.5\mathrm{Cha}o^{T-1}$, as the threshold of the sharp increase. As shown in Figures 3a, b & 4c, for 11 symptomatic subjects, only 5/11 Chao prediction was in advance of symptom appearance in comparison to the 11/11 mNFE prediction, which validated the accuracy of the mNFE method in detecting the tipping point on an individual basis. Note that similar results were obtained with other indexes of alpha-diversity, such as Shannon, Simpson and richness.

(A) Alpha-diversity (Chao index) in three groups at the overall taxonomic level. (B) Alpha-diversity (Chao index) in three groups at the genus level. (C) The alpha-diversity (Chao index) curves of 11 symptomatic individuals. (D) PCA plots comparing three groups for all individuals. (E) Comparison of Bray-Curtis distance difference for T1D individuals in different groups. (F) Comparison of Bray-Curtis distance difference for seroconversion individuals in different groups.

Similarly, the microbiota beta-diversity analysis also showed a clear separation among T1D cases with control and seroconversion groups (Figure 4d). Finally, we calculated the difference in microbial community (Bray-Ccurtis distance) between each time point and the reference time points (the first two time points) for all symptomatic individuals. In Figure 4e,f there are significant differences between before and after the tipping point detected by mNFE for both T1D or seroconversion individuals. Moreover, the difference before and after the tipping point was higher than that before and after the onset of symptoms.

### Analysis of high-frequency species of all symptomatic subjects with mNFE

#### Critical states of T1D based on high-frequency species

The DNBs varied among individuals even in the case of the same disease, but there still exist some common DNBs. We counted the common DNBs for all symptomatic individuals, and defined the high frequency species (Supplementary Table S1) as the DNBs common in two T1D individuals or in five or more out of 11 seroconverted individuals. Among them, there were 88 high-frequency species in two T1D infants and 77 high-frequency species in 11 seroconverted infants. To validate the effectiveness of mNFE score, we respectively warned the early-warning signals of seroconversion and T1D individuals based on the high-frequency species of these 11 symptomatic individuals, and the results showed that the critical states were all detected before the onset of symptoms (seen in Supplementary Material, Figure S4).

**Figure 5.**
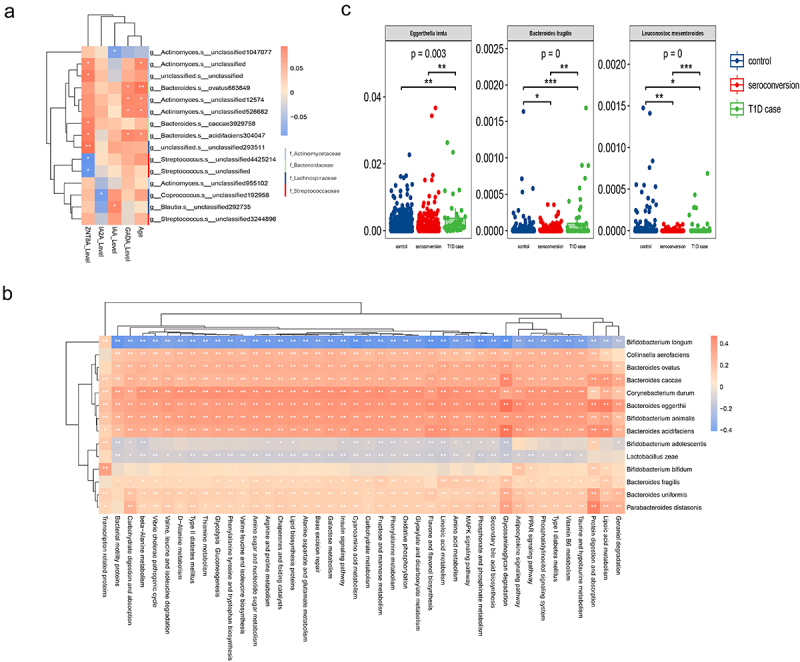
Functional analyses of high-frequency species.

T1D is an autoimmune disease characterized by immune-mediated destruction of pancreatic beta cells. GADA, IA-2A, IAA, and ZnT8A are some of the most reliable biomarkers for autoimmune diabetes T1D in both children and adults. The samples form symptomatic individuals had different clinical profiles for autoantibodies including IAA, GADA, IA-2A, and ZnT8A levels. Therefore, to prioritize gut microbial species, we determined Spearman’s correlations between the abundances of microbial high-frequency species and clinical features using all symptomatic individuals. The close relationships of gut microbial high-frequency species with autoantibodies were observed, especially for GADA and ZnT8A (Figure 5a). Previously, Bacteroides acidifaciens was reported to be a potential agent for modulating metabolic disorders such as diabetes and obesity.^34^ At the taxonomic level, the abundances of some high-frequency species, such as Bacteroides acidifaciens and Bacteroides ovatus, are closely related to the clinically important metadata of T1D, which validates the effectiveness of mNFE in identifying the DNBs.

(A) Spearman’s correlations between high-frequency species and clinical metadata. (B) Heatmap of the Spearman’s correlation coefficients between high-frequency species and microbiota pathways. (C) Relative abundance of Eggerthella lenta, Bacteroides fragilis and Leuconostoc mesenteroides. Each species corresponds to a boxplot showing the p-values for the overall differences between the groups obtained from Kruskal-Wallis nonparametric tests, as well as the markers of the differential significance levels obtained from the Dunn’s post hoc tests (* *p* < .05, **p < .01, ***p < .001).

### Functional analysis of the high-frequency species

We used PICRUSt^35^ to predict the functional potential of microbial communities and identified 247 pathways, among which we selected differential abundance pathways. To investigate whether high-frequency species are associated with the progression of T1D, we conducted Spearman’s correlation analysis (*p* < .05) between the high-frequency species and selected pathways. As shown in Figure 5b, 14 high-frequency species were significantly related to the metabolic pathways during the progression of T1D, including alanine, aspartate, glutamate metabolism, carbohydrate digestion and absorption, fructose and mannose metabolism, adipocytokine signaling pathway valine, leucine and isoleucine biosynthesis, and lipoic acid metabolism, all of which have been previously associated with insulin resistance and glucose content.^36–40^ Others pathways that are not directly associated with the blood glucose control and insulin resistance, they may actively influence a metabolic environment in the gut that is permissive to inflammation and promotes contributes to T1D pathogenesis.

In addition, literature searching also validates that some of these high-frequency species can improve blood glucose control and insulin resistance. For instance, Bacteroides uniformis and Bacteroides acidifaciens show promise as potential agents for modulating metabolic disorders such as diabetes and obesity, contributing to obesity prevention and improved insulin susceptibility in diabetes.^41,42^ Parabacteroides distasonis-derived nicotinic acid was reported as a vital bioactive molecule that fortifies intestinal barrier function by activating intestinal G-protein-coupled receptor 109a (GPR109a), leading to the amelioration of insulin resistance.^43^ Collinsella aerofaciens has a positive correlation with 2−Hydroxybutyric acid (2HB) in the original study. Due to the activation of other metabolic pathways during insulin resistance, 2-hydroxybutyric acid is also synthesized as a coproduct of protein metabolism, thus the progression of insulin resistance is intrinsically related to the increase of 2HB levels.^44^ Therefore, Collinsella aerofaciens may have an influence on increasing the 2HB levels, thereby holding predictive potential for the development of insulin resistance.

Therefore, the 14 high-frequency species likely play multiple roles in the progression of T1D, by (1) being involved in blood glucose control and insulin resistance through pathways mentioned above (and the metabolites produced); (2) pathways that may serve other functional roles in the development of T1D.

### ‘Dark species’ revealed by mNFE

In addition, we conducted a differential analysis of the microbial species and found that 70 species were differentially abundant among the three groups (p ≤ .05, Kruskal-Wallis test) (Supplementary Table S2). As shown in Figure 5c, in contrast to the T1D case group, there were significant differences in the relative abundances of some species in the control group and seroconversion group, such as Eggerthella lenta, Bacteroides fragilis and Leuconostoc mesenteroides. In particular, the relative abundance of Bacteroides fragilis and Leuconostoc mesenteroides differed significantly between the control and seroconverted groups. Twenty-five differentially abundant species are also DNBs that can facilitate the detection of critical state. We then focused on the T1D ’dark species’ (Supplementary Table S3) that were not differentially abundant in DNBs but sensitive to mNFE score. Although they are generally ignored in traditional differential abundance analysis, many of these species play important roles in T1D progression. Through a literature search, we found that some ’dark species’ are closely related to the process of diabetes. For example, Bifidobacterium adolescentis is effective in relieving diabetes and may be related to its dominant core genome and gut microbiota modulation capacity.^45^ Moreover, Bifidobacterium adolescentis was also associated with the levels of antibodies GADA and ZnT8A. Recent studies suggest that Parabacteroides distasonis could exert protective effects against certain diseases, including multiple sclerosis, type 2 diabetes, colorectal cancer, and inflammatory bowel disease. Some evidence that Parabacteroides distasonis may be involved in the pathogenesis of diabetes has begun to emerge. But there is a lack of an established consensus on Parabacteroides distasonis’ role in modulating the human gut microbiota and, more importantly, the pathogenicity of the bacterium.^46^ Similarly, Spearman correlation analysis (p < .05) was performed between ’dark species’ and microbiota KEGG pathways (Supplementary Material, Figure S5). These results showed that, although the ’dark species’ could not be significantly different between the control group and the seroconversion group compared with the T1D case group, they were significantly associated with the development of the T1D.

### Connection properties and robustness test of microbial association network

**Figure 6.**
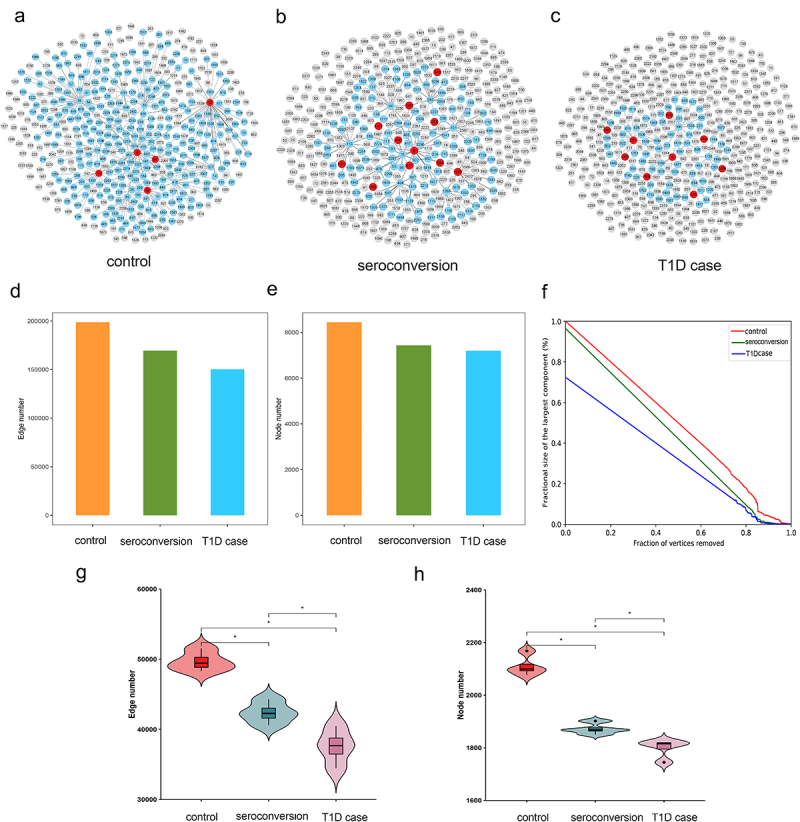
Analysis of the microbiome association network for each group.

Microbial association network analysis showed that the microbiome in the control group without seroconversion formed a more robust network than the microbiome of the seroconverted and even converted T1D case groups (Figure 6a–c). We then compared the connectivity and vulnerability of the microbial networks among these groups, i.e., the control group, seroconversion group, and T1D case group. The number of nodes and edges followed order of control > seroconversion > T1D case (Figure 6d–e). The control group had the highest numbers of edges and nodes, indicating higher connectivity of the control group microbial networks. In addition, to measure the vulnerability of these microbial networks, we simulated system collapse by sequentially removing individual nodes from the network. As shown in Figure 6f, the area under the curve of the control group was the largest, indicating that its association network was the most robust to the simulated disruption. By contrast, the association network in the T1D case group was the most vulnerable. Next, we investigated the connectivity of the microbiome network of each individual under different groups. As shown in Figure 6g,h, the groups could be ordered control > seroconversion > T1D case on the basis of node number and edge number of association network among the three groups. These two network properties indicated that network connectivity was the strongest in individuals belonging to the control group and the weakest in individuals belonging to the case group.

(A–C) The dynamic evolution of microbial association network constructed of T1D among three groups, where gray nodes represent unrelated species that are present in the other two groups, blue nodes represent species present in each corresponding group, and red nodes represent identified DNBs. (D–E) Connectivity properties (node number, edge number) of the association network of each group. (F) Fragility of the association network of each group. The y-axis shows the fragility, which means the percentage of remaining nodes in the giant (largest) component, and the x-axis shows the percentage of nodes/vertices removed. The fragility of each network characterizes the robustness. (G–H) Violin chart reflecting the connectivity property of the association network for each group of subjects, including node number and edge number.

## Discussion

It is important to characterize the critical states of diseases, such as the pre-onset stage of T1D. Detecting early-warning signals for the critical transition to the disease state may provide appropriate timing for preventing or at least preparing for catastrophic deterioration. The 16S sequence data from stool samples have rich categorical information to facilitate the detection of pre-disease state at different taxonomic levels. However, detecting the critical states of the gut microbiome in T1D is important but also difficult on a single-sample basis. In this paper, we proposed a novel method, i.e., mNFE, to detect the critical state before disease deterioration on the basis of a single sample. Based on the MB method from SPIEC-EASI, we constructed the microbial association network. By calculating the NFE on the sample-specific association network, we evaluated the criticality of each species from its local- or sub-network. Finally, global perturbation brought by each individual sample against a group of given control/reference samples was quantified based on the mNFE method. We found that the mNFE method was able to quantify network fluctuation or collective fluctuation of molecules, which is the key criterion of the tipping point, rather than the random fluctuation of molecules, thereby providing reliable early-warning signals of the critical state.

The numerical simulation validated the robustness of mNFE under different noise levels. With the increase of noise strength, mNFE can still stably provide robust early-warning signals for the upcoming tipping point. Based on real datasets of human gut microbiota, mNFE successfully identified the pre-disease states as well as their DNBs consistently from the different taxonomic levels, thereby providing more accurate and effective early-warning signals during the development of T1D. The diversity analysis and accuracy analysis verified the effectiveness and accuracy of the mNFE method. We found some high-frequency species are closely associated with the diabetes-associated autoantibodies. In addition, we revealed some ‘dark species’ that without differential abundance but were impressible to mNFE score, which also play an important role in the development of T1D. By comparing the connectivity and fragility of microbial association network between the control and case groups, we revealed that the connectivity of the microbial association network in the control group was stronger than that in the case group, but the microbial association network in the case group was more fragile. It is therefore of great potential and application prospect in personalized diagnosis.

## Conclusions

In summary, mNFE is an effective and robust method based on a single sample from each individual, which can not only detect the critical state during disease development but also identify the corresponding DNBs. More importantly, mNFE is applicable not only to T1D but also to other diseases. Thus, the proposed mNFE method is of great significance for precision diagnosis and treatment. Moreover, theoretically, any omics data (e.g., metagenomic data or metabolomics data) that can dynamically reflect changes in disease development can be used to detect the critical state or tipping point in a similar way.

## Methods

### Theoretical background

DNBs are a set of molecules (i.e., genes or proteins) or molecular modules that can reliably detect the early-warning signals of disease state transition.^47^ Specifically, when a biological system from a stable state approaches a critical state, DNB molecules satisfy three statistical conditions^8^: the correlation between any two members in the DNB group sharply increases; the correlation between any one member in the DNB group and any other non-DNB member rapidly decreases; and the standard deviation for any member in the DNB group drastically increases. These are necessary conditions for phase transition in a biological system. Briefly speaking, DNB group is a set of variables with “critical collective fluctuations” (CCF), which can quantify a pre-disease state or “WeiBing” state called in traditional Chinese medicine. CCF principle in terms of variables is also consistent with “critical slowing down” (CSD) in terms of states.^5–8,22,23^ According to theoretical results, the tipping points or critical states of many complex diseases have been successfully detected.^48–50^

The mNFE method is based on SPIEC-EASI^51^ and NFE,^52,53^ which can measure the information content of a network rather than a number of random variables (i.e. Shannon entropy), thus quantifying the potential or function of a network from a system perspective. Based on the microbial profile, a sample-specific association network is built on an individual basis. Then, the NFE is calculated according to the local network information. Finally, the mNFE is used to quantify the perturbation brought by the single sample for local- or sub-network. The global mNFE score at each tipping point is used to detect the advent of critical states and provide early-warning signals of pre-disease states.

### Algorithm to detect the tipping point of complex diseases based on mNFE

**Figure 7.**
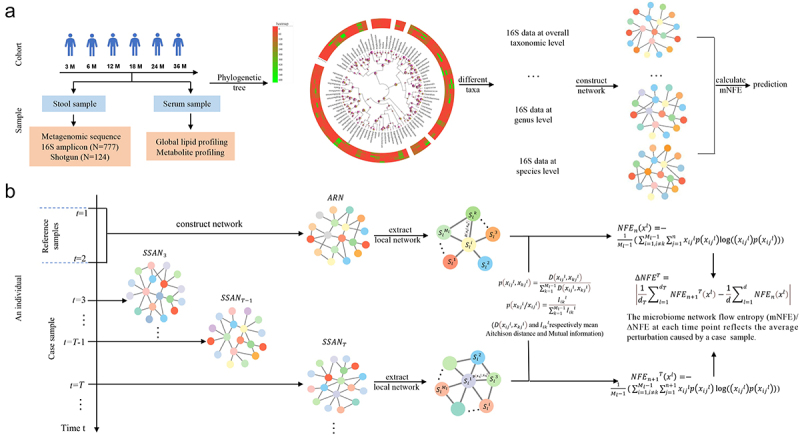
Schematic illustration of detecting the pre-disease state by using the association network based mNFE.

The mNFE is a model-free and data-driven method based on three statistical conditions detailed above.^7,8^ By calculating the NFE from these statistical conditions, mNFE provides the early-warning signals of a disease based on a single-sample and the reference samples. Note that the samples from the first two time points or the normal samples are usually taken as the reference samples. We developed the following algorithm to detect the critical state with only a single sample from each individual (Figure 7).

(A) Outline of analytical study flow. This illustrates the number of serum and stool samples collected at each time-point. Based on the collected 16S samples, we construct phylogenetic trees and extract different taxonomic data to construct the network so as to develop the mNFE algorithm. (B) By extracting the local network from the association network of the reference samples (ARN) and sample-specific association network (SSAN), we calculate the joint probability and conditional probability so as to calculate the microbiome network flow entropy (mNFE).

### [step1] construct sample-specific association network

We perform SParse InversE Covariance Estimation for Ecological Association Inference (SPIEC-EASI) to infer a microbial association network, using the neighborhood selection (mb) method with a minimum l threshold of 0.01.^54^ All steps are computed using the R package SpiecEasi.

To begin with, based on the n reference samples $\left\{s_{1},s_{2},\ldots s_{n}\right\}$, the species taxonomy matrix M includes m rows/species and n columns/samples. We transform matrix M into an association matrix AM based on SPIEC-EASI. Based on this matrix AM, the association network of the reference samples is obtained, i.e., ARN. Then, a case sample is added to the reference samples at each time point t=T, which forms the perturbed/mixed samples $\left\{s_{1},s_{2},\ldots ,s_{n},s_{\mathrm{cas}e_{T}}\right\}\;$ with the species taxonomy matrix as $M_{T}$. Similarly, with association matrix $AM_{T}$, a sample-specific association network of the perturbed/mixed samples is acquired at time point T, i.e.,$\mathrm{SSA}N_{T}.$

### [step2] extract local network from global network

Each local network $N^{l}\left(l=1,\ldots ,d\right)$ is extracted from the association network ARN of the n reference samples or the sample-specific association network $\mathrm{SSA}N_{T}$ of the n + 1 samples, where d and $d_{T}$ are the number of species in the corresponding network. Local network $N^{l}$centers on specie ${S_{l}}^{i}\left(i=1,2,\ldots ,M_{l}\right)$ and the first-order neighborhood species are the other $M_{l}-1$ species except center species.

### [step3] calculate the joint probability and conditional probability of species under n samples

For each local network $N^{l}\left(l=1,\ldots ,d\right)$, the joint probability $p\left({x_{\mathrm{ij}}}^{l},{x_{\mathrm{kj}}}^{l}\right)$ of the center specie ${S_{l}}^{i}\left(i=1,2,\ldots ,M_{l}\right)$ and neighborhood specie ${S_{l}}^{k}\left(k=1,\ldots i-1,i+1,\ldots ,M_{l}\right)$ based on n reference samples $\left\{s_{1},s_{2},\ldots ,s_{n}\right\}$ is defined by

$$p\left({x_{\mathrm{ij}}}^{l},{x_{\mathrm{kj}}}^{l}\right)=\frac{D\left({x_{\mathrm{ij}}}^{l},{x_{\mathrm{kj}}}^{l}\right)}{\sum_{k=1}^{M_{l}-1}D\left({x_{\mathrm{ij}}}^{l},{x_{\mathrm{kj}}}^{l}\right)},$$

where $D\left({x_{\mathrm{ij}}}^{l},{x_{\mathrm{kj}}}^{l}\right)$ is the Aitchison distance of the center specie ${S_{l}}^{i}\left(i=1,2,\ldots ,M_{l}\right)$ and neighborhood specie ${S_{l}}^{k}\left(k=1,\ldots i-1,i+1,\ldots ,M_{l}\right)$ based on n reference samples, calculated as follows:

$$D\left({x_{\mathrm{ij}}}^{l},{x_{\mathrm{kj}}}^{l}\right)=\left|\log\left(\frac{{x_{\mathrm{ij}}}^{l}}{S\left({x_{i}}^{l}\right)}\right)-\log\left(\frac{{x_{\mathrm{kj}}}^{l}}{S\left({x_{k}}^{l}\right)}\right)\right|,$$

with

$$S\left({x_{i}}^{l}\right)={\left(\overset{n}{\underset{j=1}{\prod }}{x_{\mathrm{ij}}}^{l}\right)}^{1/n},$$

$$S\left({x_{k}}^{l}\right)={\left(\overset{n}{\underset{j=1}{\prod }}{x_{\mathrm{kj}}}^{l}\right)}^{1/n},$$

where ${x_{\mathrm{ij}}}^{l}$ and ${x_{\mathrm{kj}}}^{l}\;$are the taxonomies of species ${S_{l}}^{i}$ and ${S_{l}}^{k}$of sample j, respectively.

And the conditional probability $p\left({x_{\mathrm{kj}}}^{l}/{x_{\mathrm{ij}}}^{l}\right)$ of neighborhood specie ${S_{l}}^{k}\left(k=1,\ldots i-1,i+1,\ldots ,M_{l}\right)$ with respect to center specie ${S_{l}}^{i}\left(i=1,2,\ldots ,M_{l}\right)$ is as follows:

$$p\left({x_{\mathrm{kj}}}^{l}/{x_{\mathrm{ij}}}^{l}\right)=\frac{{I_{\mathrm{ik}}}^{l}}{\sum_{k=1}^{M_{l}-1}{I_{\mathrm{ik}}}^{l}},$$

where ${I_{\mathrm{ik}}}^{l}$ is the mutual information of the center specie ${S_{l}}^{i}\left(i=1,2,\ldots ,M_{l}\right)$ and neighborhood specie ${S_{l}}^{k}\left(k=1,\ldots i-1,i+1,\ldots ,M_{l}\right)$ under n reference samples.

### [step4] calculate network flow entropy (NFE) of species under n samples

For each local network $N^{l}\left(l=1,\ldots ,d\right)$, the NFE of species ${S_{l}}^{i}\left(i=1,\ldots ,M_{l}\right)$ based on n reference samples is defined as:

$$\mathrm{NF}E_{n}\left(x^{l}\right)=-\frac{1}{M_{l}-1}(\overset{M_{l}-1}{\underset{i=1,i\neq k}{\sum }}\overset{n}{\underset{j=1}{\sum }}{x_{\mathrm{ij}}}^{l}p\left({x_{\mathrm{ij}}}^{l}\right)\log\left(\left({x_{\mathrm{ij}}}^{l}\right)p\left({x_{\mathrm{ij}}}^{l}\right)\right)$$

with

$$p\left({x_{\mathrm{ij}}}^{l}\right)=\frac{p\left({x_{\mathrm{ij}}}^{l},{x_{\mathrm{kj}}}^{l}\right)}{p\left({x_{\mathrm{kj}}}^{l}/{x_{\mathrm{ij}}}^{l}\right)}.$$

### [step5] calculate network flow entropy (NFE) of species under n + 1 samples

For each local network $N^{l}\left(l=1,\ldots ,d\right)$ at a time point T, we repeat the previous processes for the mixed n + 1 samples, and the NFE of species ${S_{l}}^{i}\left(i=1,\ldots ,M_{l}\right)$ based on n + 1 samples is as follows:

$$\mathrm{NF}{E_{n+1}}^{T}\left(x^{l}\right)=-\frac{1}{M_{l}-1}(\sum_{i=1,i\neq k}^{M_{l}-1}\sum_{j=1}^{n+1}{x_{\mathrm{ij}}}^{l}p\left({x_{\mathrm{ij}}}^{l}\right)\log\left(\left({x_{\mathrm{ij}}}^{l}\right)p\left({x_{\mathrm{ij}}}^{l}\right)\right)$$

### [step6] calculate the microbiome network flow entropy (mNFE)

The average disturbance network flow entropy (NFE) $\Delta \mathrm{NF}E^{T}$ of species is calculated by

$$\Delta \mathrm{NF}E^{T}=\left|\frac{1}{d_{T}}\overset{d_{T}}{\underset{l=1}{\sum }}\mathrm{NF}{E_{n+1}}^{T}\left(x^{l}\right)-\frac{1}{d}\overset{d}{\underset{l=1}{\sum }}\mathrm{NF}E_{n}\left(x^{l}\right)\right|$$

The ∆NFE score at each time point reflects the average perturbation caused by a case sample, it can also be called the Microbiome network flow entropy (mNFE).

## Supplementary Material

Supplementary Table S3.xlsx

Supplementary Materials.docx

Supplementary Table S2.xlsx

Supplementary Table S1.xlsx

## Data Availability

Metagenomic sequencing data of T1D^33^ can be downloaded from https://diabimmune.broadinstitute.org/diabimmune/(NCBI BioProject ID: PRJNA231909). To ensure reproducible results, the original code is available at https://github.com/G-R-0/mNFE.
