# Supplementary material for: mNFE: microbiome network flow entropy for detecting pre-disease states of type 1 diabetes
Source: Gut Microbes. 2024 Mar 21;16(1):2327349. doi: 10.1080/19490976.2024.2327349 (PMC10962612; doi:10.1080/19490976.2024.2327349)
Supplement: Supplementary Materials.docx [file KGMI_A_2327349_SM7908.docx]

**mNFE: Microbiome network flow entropy for detecting pre-disease states of type 1 diabetes Supplementary Material:**

**Contents**

**A. The details of numerical simulation** .............................................................................................................................................. S1

**B. The detection of tipping point for seroconversion and T1D based on mNFE** **at the genus level and species level**..............................................................................................................................................................................................................S3

**References**……………………………………………………………………………………....……………………………….……………........ S9

1. **The details of numerical simulation**

A regulatory network with 8 genes (Figure S1) was used to conduct a numerical simulation for detecting the pre-disease state using mNFE. Such molecular regulatory networks are often used to study various gene regulatory activities including transcription and translation^1,2^, and cyclic reaction^3^ and nonlinear biological processes^4,5,6^. The following 8 differential equations represent the gene regulations of 8 genes in the network. In the network, gene regulation is represented in a Michaelis-Menten form with the degradation rates, which are linearly proportional to the concentrations of the corresponding genes.


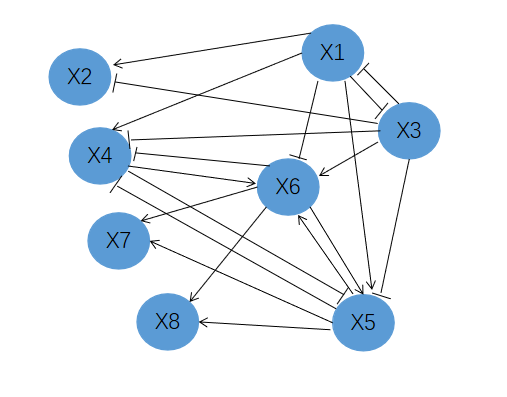


**Figure S1.** A model of an eight-molecular network. In this sketch of a molecular network, there are eight nodes whose dynamical regulatory relationships are given as stochastic system Eq.(S1). The edges represent positive or negative regulations among nodes.

$$\begin{matrix} \frac{dz_{1}(t)}{dt}=\frac{\left|P\right|-3}{8}+\frac{3-\left|P\right|}{8(1+z_{3}(t))}-\frac{3+\left|P\right|}{8}z_{1}(t)+\xi_{1}(t) \\ \frac{dz_{2}(t)}{dt}=\frac{\left|P\right|-2}{8}+\frac{(2-\left|P\right|)z_{1}(t)}{8(1+z_{1}(t))}+\frac{2-\left|P\right|}{8(1+z_{3}(t))}-\frac{1}{2}z_{2}(t)+\xi_{2}(t) \\ \frac{dz_{3}(t)}{dt}=\frac{\left|P\right|-3}{8}+\frac{3-\left|P\right|}{8(1+z_{1}(t))}-\frac{3+\left|P\right|}{8}z_{3}(t)+\xi_{3}(t) \\ \frac{dz_{4}(t)}{dt}=\frac{\left|P\right|-9}{8}+\frac{(5-\left|P\right|)z_{1}(t)}{8(1+z_{1}(t))}+\frac{5-\left|P\right|}{8(1+z_{3}(t))}+\frac{1}{4(1+z_{5}(t))}+\frac{1}{4(1+z_{6}(t))}-\frac{5}{4}z_{4}(t)+\xi_{4}(t) \\ \frac{dz_{5}(t)}{dt}=-\frac{3}{8}\text{+}\frac{z_{1}(t)}{8(1+z_{1}(t))}+\frac{1}{8(1+z_{3}(t))}+\frac{1}{4(1+z_{4}(t))}+\frac{z_{6}(t)}{4(1+z_{6}(t))}-\frac{5}{4}z_{5}(t)+\xi_{5}(t) \\ \frac{dz_{6}(t)}{dt}=-\frac{1}{8}+\frac{1}{8(1+z_{1}(t))}\text{+}\frac{z_{3}(t)}{8(1+z_{3}(t))}\text{+}\frac{z_{4}(t)}{4(1+z_{4}(t))}\text{+}\frac{z_{5}(t)}{4(1+z_{5}(t))}-\frac{5}{4}z_{6}(t)+\xi_{6}(t) \\ \frac{dz_{7}(t)}{dt}=\frac{3z_{5}(t)}{4(1+z_{5}(t))}\text{+}\frac{3z_{6}(t)}{4(1+z_{6}(t))}-\frac{7}{4}z_{7}(t)+\xi_{7}(t) \\ \frac{dz_{8}(t)}{dt}=\frac{z_{5}(t)}{(1+z_{5}(t))}+\frac{z_{6}(t)}{(1+z_{6}(t))}-2z_{8}(t)+\xi_{8}(t) \end{matrix}$$

$\left( S \right.\left. 1 \right)$

where $p$ is a scalar control parameter and $\varsigma_{i}(t)(i = 1, 2, ..., 8)$ are Gaussian noises with zero means and covariances $k_{ij}=Cov(\varsigma_{i},\varsigma_{j})$. $z_{i}(t)(i = 1, 2, ..., 8)$ represent the concentrations of mRNA-$i$. In Eq.(S1), there is the degradation rates of mRNAs $\text{R=(}\frac{\text{3+}\left|P\right|}{8}\text{，}\frac{1}{2}\text{，}\frac{\text{3+}\left|P\right|}{8}\text{，}\frac{5}{4}\text{，}\frac{5}{4}\text{，}\frac{5}{4}\text{，}\frac{7}{4}\text{，}2)$. The stable equilibrium point of the differential equations Eq.(S1) is $\overline{Z}=(\overline{z_{1}},\overline{z_{2}},\overline{z_{3}},\ldots,\overline{z_{8}})=(0,0,0,\ldots,0)$. The differential equations Eq.(S1) can be transformed into the difference equations $Z(k+1)=f(Z(k),p)$using the Euler scheme with a short time interval $\Delta t$. The result is as follows:

$\begin{matrix} z_{1}(k+1)=z_{1}(k)+\left[\frac{\left|P\right|-3}{8}+\frac{3-\left|P\right|}{8(1+z_{3}(t))}-\frac{3+\left|P\right|}{8}z_{1}(t)+\xi_{1}(t)\right]\Delta k \\ z_{2}(k+1)=z_{2}(k)+\left[\frac{\left|P\right|-2}{8}+\frac{(2-\left|P\right|)z_{1}(t)}{8(1+z_{1}(t))}+\frac{2-\left|P\right|}{8(1+z_{3}(t))}-\frac{1}{2}z_{2}(t)+\xi_{2}(t)\right]\Delta k \\ z_{3}(k+1)=z_{3}(k)+\left[\frac{\left|P\right|-3}{8}+\frac{3-\left|P\right|}{8(1+z_{1}(t))}-\frac{3+\left|P\right|}{8}z_{3}(t)+\xi_{3}(t)\right]\Delta k \\ z_{4}(k+1)=z_{4}(k)+\left[\frac{\left|P\right|-9}{8}+\frac{(5-\left|P\right|)z_{1}(t)}{8(1+z_{1}(t))}+\frac{5-\left|P\right|}{8(1+z_{3}(t))}+\frac{1}{4(1+z_{5}(t))}+\frac{1}{4(1+z_{6}(t))}-\frac{5}{4}z_{4}(t)+\xi_{4}(t)\right]\Delta k \\ z_{5}(k+1)=z_{5}(k)+\left[-\frac{3}{8}\text{+}\frac{z_{1}(t)}{8(1+z_{1}(t))}+\frac{1}{8(1+z_{3}(t))}+\frac{1}{4(1+z_{4}(t))}+\frac{z_{6}(t)}{4(1+z_{6}(t))}-\frac{5}{4}z_{5}(t)+\xi_{5}(t)\right]\Delta k \\ z_{6}(k+1)=z_{6}(k)+\left[-\frac{1}{8}+\frac{1}{8(1+z_{1}(t))}\text{+}\frac{z_{3}(t)}{8(1+z_{3}(t))}\text{+}\frac{z_{4}(t)}{4(1+z_{4}(t))}\text{+}\frac{z_{5}(t)}{4(1+z_{5}(t))}-\frac{5}{4}z_{6}(t)+\xi_{6}(t)\right]\Delta k \\ z_{7}(k+1)=z_{7}(k)+\left[\frac{3z_{5}(t)}{4(1+z_{5}(t))}\text{+}\frac{3z_{6}(t)}{4(1+z_{6}(t))}-\frac{7}{4}z_{7}(t)+\xi_{7}(t)\right]\Delta k \\ z_{8}(k+1)=z_{8}(k)+\left[\frac{z_{5}(t)}{(1+z_{5}(t))}+\frac{z_{6}(t)}{(1+z_{6}(t))}-2z_{8}(t)+\xi_{8}(t)\right]\Delta k \end{matrix}$ (S2)

It is easy to note that $Z(k)$ is the vector of $Z(t)$ at the time instant $k\Delta t$. The Jacobian matrix of Eq.(S2) can be defined as $\left. J=\frac{\partial f(Z(k);p)}{\partial Z} \right|_{Z=\overline{Z}}$, where

$J=e^{\Delta t\bullet A}$

$\left( S3 \right)$

With

$A=\left[\begin{matrix} -\frac{3+\left|P\right|}{8} & 0 & \frac{\left|P\right|-3}{8} & 0 & 0 & 0 & 0 & 0 \\ \frac{3+\left|P\right|}{8} & -\frac{1}{2} & \frac{3+\left|P\right|}{8} & 0 & 0 & 0 & 0 & 0 \\ \frac{3+\left|P\right|}{8} & 0 & \frac{3+\left|P\right|}{8} & 0 & 0 & 0 & 0 & 0 \\ \frac{3+\left|P\right|}{8} & 0 & \frac{3+\left|P\right|}{8} & -\frac{5}{4} & -\frac{1}{4} & -\frac{1}{4} & 0 & 0 \\ \frac{1}{8} & 0 & -\frac{1}{8} & -\frac{1}{4} & -\frac{5}{4} & \frac{1}{4} & 0 & 0 \\ -\frac{1}{8} & 0 & \frac{1}{8} & \frac{1}{4} & \frac{1}{4} & -\frac{5}{4} & 0 & 0 \\ 0 & 0 & 0 & 0 & \frac{3}{4} & \frac{3}{4} & -\frac{7}{4} & 0 \\ 0 & 0 & 0 & 0 & 1 & 1 & 0 & -2 \end{matrix}\right]$

From Eq.(S3), by taking $\Delta t=1$, we can obtain eight distinct eigenvalues ($0.78|P|，0.61，0.47，0.37，0.27，0.22，0.17，0.14$) by taking $\Delta t=1$. It is obvious that the largest eigenvalue ${0.78}^{|p|}$→1 when $p$→0. Therefore, the equilibrium point $\overline{Z}$ is stable when $p\in(0,1]$ and $p=0$ is a bifurcation point, at which the system undergoes a critical transition. We aimed to detect early-warning signals that indicate the critical transition as a control parameter $p$ approaches a bifurcation point 0. According to mNFE method, for each simulation trial, we used the 28 samples generated when the control parameter $p$ was far away from the bifurcation point $p$ = 0 (e.g.,$p\in[-0.50, 0.25]$) as the reference samples. Then in each simulation trial, based on the single sample $\{z_{1},z_{2}, \ldots, z_{28}\}$ derived for each parameter value $p\in[-0.5, 0.25]$, the sNFE score was calculated following the mNFE method (see Method section in the main text), as shown in Figure 2 in the main text.

**B. The detection of tipping point for seroconversion and T1D based on mNFE at the genus level and species level**

In order to improve the effectiveness of the mNFE method, we detected the early-warning signals of seroconversion and T1D at the genus and species levels, respectively. As shown in the Figure S2A and Figure S3A, the mNFE scores sharply increase for symptomatic subjects, but not for asymptomatic subjects. It means that the early-warning signals can be effectively detected by our mNFE method. Figure S2B-C and Figure S3B-C show that the mNFE scores for eleven symptomatic subjects can correctly detect the early-warning signals before the onset of seroconversion and T1D. Although the tipping points detected at the genus and species levels were somewhat different from those detected at the overall level, both were detected before the onset of seroconversion and T1D, thereby improving the effectiveness and accuracy of the mNFE method.





**Figure S2.** The identification of tipping point for seroconversion and T1D based on mNFE at the genus level. (A) Line chart of mNFE scores for all 33 subjects at the genus level. The red curve stands for the mNFE scores for two T1D subjects. The green curve represents the mNFE score for nine seroconverters. The blue curve represents the mNFE score for twenty-two non-converters. (B)The individual mNFE score curves of two T1D subjects at the genus level. (C)The individual mNFE score curves of nine seroconverters at the genus level. For each mNFE curve, the yellow circle represents the upcoming seroconversion state detected by the mNFE score, and the green square represents the time point at which the onset of seroconversion symptom. The purple circle stands for the tipping point when mNFE score signal arisses, and the blue

square mark denotes the time point at which the initial T1D symptoms appears.

**Figure S3.** The identification of tipping point for seroconversion and T1D based on mNFE at the species level. (A) Line chart of mNFE scores for all 33 subjects at the species level. The red curve are mNFE scores for two T1D subjects, green curve are mNFE score for nine seroconverters subjects and blue ones for other asymptomatic subjects. (B)The individual mNFE score curves of two T1D subjects. (C)The individual mNFE score curves of nine seroconverters subjects at the species level. Each sub-curve shows the mNFE score for each symptomatic subject during the progression of the seroconversion and T1D. The yellow circle represents the predicted seroconversion state identified by the mNFE score, whereas the green square indicates the clinical diagnosis time point at which the onset of seroconversion symptom. The purple circle represents the critical states detected by the mNFE score, and the blue square mark denotes the time point when T1D onset is clinically observed.


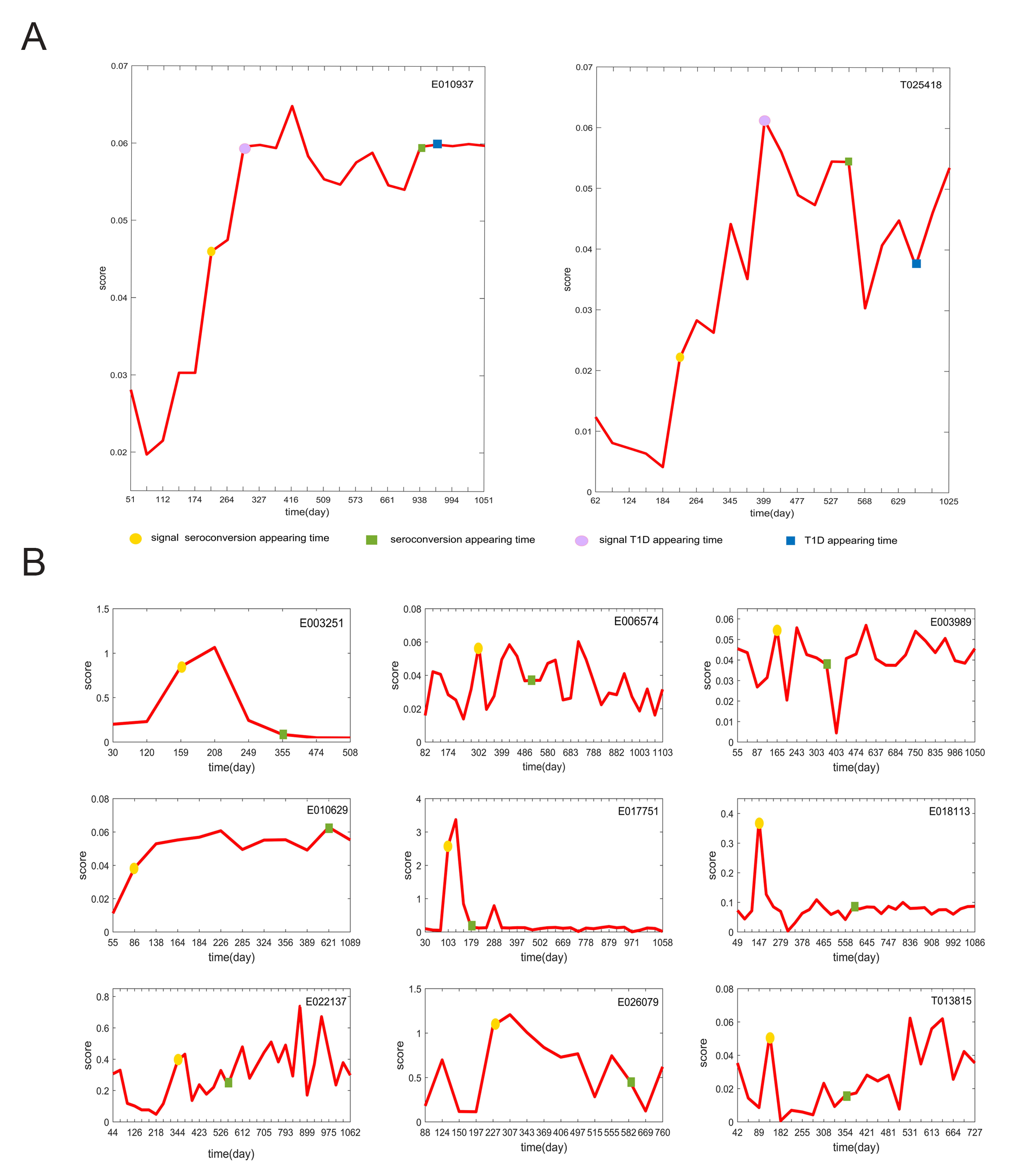


**Figure S4.** The mNFE score curves of seroconversion and T1D individuals based on high-frequency species. (A)The timing of seroconversion and T1D occurred in two TID individuals were correctly identification. (B)All seroconverted individuals were correctly detected at the time the seroconversion appeared.


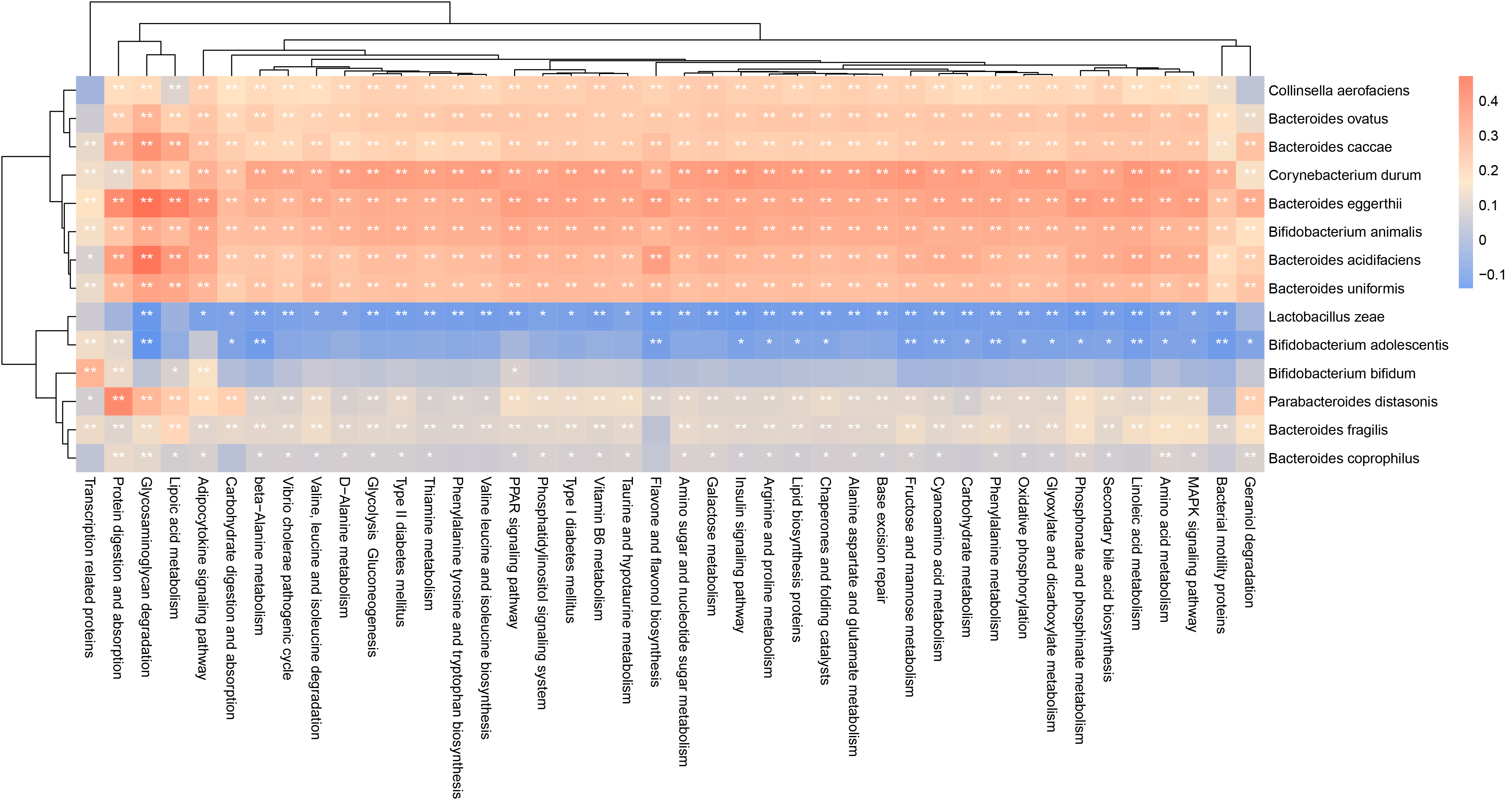


**Figure S5.** Heatmap of the Spearman’s correlation coefficients between ‘dark species’ and microbiota KEGG pathways.

**References**

1. Khanin R, Vinciotti V, Mersinias V, et al. Statistical Reconstruction of Transcription Factor Activity Using Michaelis–Menten Kinetics. Biometrics. 2007; 63:816-823.
2. Ronen M, Rosenberg R, Shraiman B, et al. Assigning numbers to the arrows: parameterizing a gene regulation network by using accurate expression kinetics. Proc Natl Acad Sci U S A. 2002; 99(16):10555-10560.
3. Sueyoshi C, Naka T. Stability Analysis for the Cellular Signaling Systems Composed of Two Phosphorylation-Dephosphorylation Cyclic Reactions. Computational Molecular Bioscience. 2017; 7:33-45.
4. Chen L, Wang R, Li C, et al. Modeling Biomolecular Networks in Cells: Structures and Dynamics. Springer London. 2010.
5. Chen L, Wang R, Zhang X. Biomolecular Networks: Methods and Applications in Systems Biology. John Wiley & Sons Inc. 2009; DOI: 10.1002/9780470488065.
6. Chen P, Li Y, Liu X, et al. Detecting the tipping points in a three-state model of complex diseases by temporal differential networks. J Transl Med. 2017; 15(1):217.
